# Radical Oxygen Species, Oxidized Low-Density Lipoproteins, and Lectin-like Oxidized Low-Density Lipoprotein Receptor 1: A Vicious Circle in Atherosclerotic Process

**DOI:** 10.3390/antiox13050583

**Published:** 2024-05-09

**Authors:** Marco Munno, Alice Mallia, Arianna Greco, Gloria Modafferi, Cristina Banfi, Sonia Eligini

**Affiliations:** 1 Unit of Functional Proteomics, Metabolomics and Network Analysis, Centro Cardiologico Monzino, 20138 Milan, Italy; marco.munno@cardiologicomonzino.it (M.M.); alice.mallia@cardiologicomonzino.it (A.M.); arianna.greco@cardiologicomonzino.it (A.G.); gloria.modafferi@cardiologicomonzino.it (G.M.); sonia.eligini@cardiologicomonzino.it (S.E.); 2 Dipartimento di Biologia e Biotecnologie “Lazzaro Spallanzani”, Università di Pavia, 27100 Pavia, Italy

**Keywords:** radical oxygen species, ox-LDL, LOX-1, atherosclerosis

## Abstract

Atherosclerosis is a complex condition that involves the accumulation of lipids and subsequent plaque formation in the arterial intima. There are various stimuli, cellular receptors, and pathways involved in this process, but oxidative modifications of low-density lipoprotein (ox-LDL) are particularly important in the onset and progression of atherosclerosis. Ox-LDLs promote foam-cell formation, activate proinflammatory pathways, and induce smooth-muscle-cell migration, apoptosis, and cell death. One of the major receptors for ox-LDL is LOX-1, which is upregulated in several cardiovascular diseases, including atherosclerosis. LOX-1 activation in endothelial cells promotes endothelial dysfunction and induces pro-atherogenic signaling, leading to plaque formation. The binding of ox-LDLs to LOX-1 increases the generation of reactive oxygen species (ROS), which can induce LOX-1 expression and oxidize LDLs, contributing to ox-LDL generation and further upregulating LOX-1 expression. This creates a vicious circle that is amplified in pathological conditions characterized by high plasma levels of LDLs. Although LOX-1 has harmful effects, the clinical significance of inhibiting this protein remains unclear. Further studies both in vitro and in vivo are needed to determine whether LOX-1 inhibition could be a potential therapeutic target to counteract the atherosclerotic process.

## 1. Introduction

Atherosclerosis is a disease of the arteries that is chronic and inflammatory in nature. It occurs when lipids are deposited on the vessel wall, leading to the thickening of the intima and the formation of a fatty streak. This accumulation of lipids and recruitment of inflammatory cells at the lesion site result in the development of an atherosclerotic plaque that grows inside the arteries, impairing their function [1].

This process occurs through a sequence of cellular and molecular events characterized by the deposition of low-density lipoproteins (LDLs) into the intima of atheroprone sites of arteries, expression of adhesion molecules [2], recruitment of circulating mononuclear cells to the endothelium with local activation of leukocytes [3,4], infiltration of monocytes, and subsequent differentiation into macrophages. Macrophages, through scavenger receptors (SRs), internalize native and modified lipoproteins [5], becoming cholesterol-rich foam cells [6] that, with lipids and inflammatory cells, contribute to atheroma development [7].

The subendothelial retention of LDLs represents the Initial event in the formation of the atherosclerotic plaque, as the LDLs trapped within the vessel wall can undergo several modifications in their size, density, and chemical properties. In particular, oxidation is the major process involved in the atherosclerotic process [8], and although the formation of oxidized low-density lipoproteins (ox-LDLs) in vivo is poorly understood, several studies have demonstrated the link between ox-LDLs and atherosclerosis onset [9]. The internalization of ox-LDLs leads to an increase in the generation of reactive oxygen species (ROS), such as superoxide anion (O_2_^−^) and hydrogen peroxide (H_2_O_2_) [10], which cause inflammation and endothelial dysfunction. Indeed, following the generation of ROS, oxidative activation promotes and contributes to the endothelial cells’ (ECs) phenotypical changes that determine the initiation of the atherosclerotic lesion [11,12,13].

The internalization of ox-LDLs by macrophages involves binding to several SRs, including SR-A, SR-BI, CD36, and the lectin-like oxidized low-density lipoprotein receptor 1 (LOX-1) [14].

## 2. Oxidative Stress and ROS Generation in Cardiovascular Diseases

Oxidative stress refers to an imbalance between the body’s production of ROS and its antioxidant defense system. ROS production occurs mainly in mitochondria during normal aerobic metabolism as well as by various enzymes like NADPH oxidase [15]. ROSs play an important role in many biological processes and can either contribute to normal processes or trigger pathological responses, depending on the cell type and source of generation. ROSs are crucial for maintaining vascular function and play a critical role in modulating the growth, proliferation, and migration of endothelial and smooth muscle cells, permeability, angiogenesis, and host defense [16]. However, excessive levels of ROSs can lead to structural changes in DNA, lipids, and proteins, altering their function. While physiological levels of ROSs are necessary for maintaining cellular signaling and homeostatic function, high levels of ROSs can trigger inflammatory signaling, metabolic dysfunction, and oxidative damage. Several diseases, such as diabetes mellitus, atherosclerosis, and stroke, are linked to an altered redox balance and oxidative stress [17].

In situations where there is oxidative stress, ROS can have a negative impact on endothelial function. Elevated ROS levels can decrease the amount of bioavailable NO, leading to a reduction in vasoprotective NO-mediated signaling. This can result in the production of peroxynitrite, a powerful oxidant that can cause the uncoupling of endothelial NO synthase (eNOS), which becomes a dysfunctional pro-oxidant enzyme [18]. Additionally, ROSs can contribute to changes in the thickness of the vasculature’s wall and lumen diameter, which increases systemic vascular resistance and can contribute to the onset and progression of atherosclerosis [19,20,21]. ROS-induced oxidative damage also activates inflammasome sensor nod-like receptor protein (NLRP) 3. This multiprotein complex triggers the secretion of the inflammatory cytokines interleukin (IL)-1β and IL-18 through the activation of the CLOCK gene and caspase-1, leading to the activation of the inflammatory cascade and ultimately resulting in pyroptosis and cellular death [22]. Although further studies are needed to determine the precise role of the NLR3 inflammasome in the atherosclerotic process, in vitro studies suggest that cholesterol crystals activate the NLRP3 inflammasome and induce lysosomal damage [23,24]. Moreover, the atherosclerotic area in the aortas of mice that lack NLRP3 components in their bone marrow was significantly smaller compared to wild-type bone-marrow mice [23].

ROSs are also integral to the proper functioning of the heart, aiding in its development, differentiation, and proliferation of cardiomyocytes, as well as in excitation–contraction coupling. However, in certain cardiac conditions, such as cardiac hypertrophy, heart failure, and ischemia–reperfusion injury, the ROS production in cardiomyocytes can become dysregulated [25]. Cardiac hypertrophy is a complex adaptative response of the heart to the increased workload, and ROSs can activate several signaling pathways involved in this disease [26]. Furthermore, ROSs can oxidize the myofibrillar proteins, leading to cardiac contractility dysfunction, observed in heart failure [27]. ROSs also play a crucial role in the setting of acute myocardial infarction and reperfusion injury. During ischemia–reperfusion injury, cell death is characterized by an accumulation of oxidized lipids and proteins resulting from an excessive ROS generation derived from an increase in NADPH oxidase activity and activation of the redox-sensitive kinases ERK and JNK [28]. The activation of these signaling pathways contributes to inducing mitochondrial oxidase dysfunction, leading to additional ROS production and Ca^2+^ overload, which are key contributors to cardiomyocyte death and myocardial injury after ischemia and reperfusion [26].

## 3. Oxidized Low-Density Lipoproteins

Lipoproteins are small particles composed of both lipids and proteins that are responsible for lipid transport in the bloodstream and their delivery to tissues.

LDLs are the primary carriers of cholesterol, containing cholesterol ester as their primary lipid component, along with some free cholesterol. ApoB-100 is the primary apolipoprotein found in LDLs. LDLs have a spherical structure with a diameter of approximately 22 nm and a density that ranges from 1.019 to 1.063 g/mL [29]. These particles are heterogeneous in size, density, and composition, and depending on the methodology used, from 2 to 38 subfractions have been identified [30]. The composition of LDL subfractions, as well as their distribution, differs between individuals and is determined by both genetic and environmental factors [31]. In particular, it has been shown that the prevalence of small, dense LDLs was associated with an increased risk for cardiovascular diseases [30,32]. In accordance, patients with cardiovascular diseases showed higher levels of small, dense LDLs compared to control subjects [32]. There are several mechanisms that have been proposed to explain how small dense LDLs may promote atherosclerosis, with the susceptibility to oxidation playing an important role [33].

In physiological conditions, our body maintains redox homeostasis. However, as a result of an increase in ROS production, several constituents of LDLs, including phospholipids, cholesterol esters, and polyunsaturated fatty acids are susceptible to oxidation, resulting in the formation of ox-LDLs [34,35]. Moreover, small, dense LDLs, due to reduced levels of antioxidants, such as vitamin E and ubiquinol-10, and increased levels of polyunsaturated fatty acids including arachidonic acid, are more susceptible to oxidation than large LDLs [31].

Numerous studies have utilized ox-LDLs in vitro prepared as a model of circulating ox-LDLs in atherosclerosis research. To induce LDL oxidation, metallic ions like copper or iron, as well as radical initiators, have been employed. However, copper-induced ox-LDLs are severely modified and highly oxidized and are not found in circulation despite being easily reproducible and prepared. Hence, minimally modified LDLs (MM-LDLs) have been developed through mild oxidation conditions and dialysis [36]. Although these LDLs display only a weak increase in thiobarbituric acid reactive substances (TBARS), they can still activate several responses in vascular cells [37]. MM-LDLs do not bind well with macrophage SRs, yet they can boost the expression of SRs, like CD36, SR-A, and macrosialin, leading to the generation of foam cells and the progression of atherosclerosis [38]. MM-LDLs result from the oxidative modification of only the lipid moiety of LDL, mainly phospholipids, without affecting the apolipoprotein ApoB-100. The oxidation of ApoB-100 occurs later, with the generation of highly ox-LDLs [39]. In particular, it has been shown that oxidized 1-palmitoyl-2-arachidonoyl-sn-glycero-3-phosphorylcholine (ox-PAPC), a crucial component of MM-LDL, is able to mimic many of the biological effects induced by MM-LDL and mildly oxidized LDL [40,41,42].

Both highly oxidized LDLs and MM-LDLs have been detected in atherosclerotic lesions, as well as in plasma [43,44]. While the levels of ox-LDLs in the plaque are significantly higher than those in circulation, circulating ox-LDL levels are very low. As ox-LDLs have a short half life in the plasma compartment and are quickly cleared by the reticuloendothelial system, the small amounts detectable in plasma are mainly MM-LDLs that show a longer half-life and can accumulate in the circulatory stream [44].

The oxidation of LDLs primarily occurs within the vessel wall, and multiple factors can contribute to the creation of ox-LDLs. These factors include the number and size of LDLs, their composition and susceptibility to oxidation, the presence of oxidative stress factors, and endothelial dysfunction [43]. Moreover, it has also been suggested that ox-LDL can move from atherosclerotic plaque to circulation, as temporal changes in ox-LDL levels have been observed in certain conditions [44]. Studies have shown a transient increase in ox-LDL plasma levels after myocardial infarction and percutaneous transluminal coronary angioplasty [45,46,47,48], indicating that ox-LDLs generated in the vascular wall can be released into the circulation when the plaque ruptures [49].

Although many aspects of the role of ox-LDLs in vivo are still unclear, and it is still unknown how and when LDLs are oxidized, increasing evidence suggests that ox-LDLs are a useful marker of cardiovascular disease. Several in vivo studies have reported high levels of ox-LDLs in patients with cardiovascular diseases, diabetes mellitus, and subjects receiving hemodialysis compared to control subjects [50,51,52,53]. Additionally, several studies have shown the association between ox-LDL levels and subclinical atherosclerosis by measuring the intima-media thickness of the carotid or femoral arteries [54,55,56,57] and coronary calcification [58].

Levels of ox-LDLs have been found to be a predictor of acute ischemic events in both seemingly healthy [59] and coronary syndrome patients [60]. Research has shown a positive correlation between ox-LDL levels and the severity of coronary disease, indicating that high levels of ox-LDL may contribute to destabilizing the atherosclerotic plaque. This destabilization can have an impact on the plaque’s composition, increasing the inflammatory process and leading to surface thrombosis [60].

Furthermore, high plasma levels of ox-LDLs have been linked to mortality and morbidity in patients with chronic congestive heart failure [61]. An in vivo study has found that plasma levels of ox-LDL are associated with a decrease in cardiac function, independent of vascular impairment [62].

## 4. Oxidized High-Density Lipoproteins

It is widely known that high-density lipoproteins (HDL) play a protective role in preventing various cardiovascular diseases, such as coronary artery disease and atherosclerosis [63,64]. However, recent studies have shown that HDLs can also undergo oxidative modifications, just like LDLs do, which can modify their anti-atherogenic properties. [65,66]. In accordance, it has been shown that the in vitro oxidation of HDLs with copper or acrolein reduced their cholesterol transport activity [67,68] and diminished the capacity to induce cellular cholesterol efflux by the membrane-associated ATP-binding cassette transporter A1 (ABCA1) [66]. The oxidation of HDLs is mainly due to the oxidation of ApoA-I, which is the major protein component of HDLs. Malondialdehyde and myeloperoxidase target ApoA-I, leading to the oxidation of the Met112 residue [69]. This oxidation reduces the lipid hydroperoxides inactivating capacity and the covalent modifications induced by reactive carbonyl, resulting in a significant impairment of the cholesterol efflux. [66,70]. Indeed, increased levels of ox-HDLs have been measured in several cardiovascular diseases, and a correlation between ox-HDL levels and coronary artery calcification progression has been observed [71]. In addition, a marked association has been shown between high-risk coronary plaque features and progression and oxidized lipoproteins including ox-LDL and ox-HDL [72].

The presence of ox-HDLs has been detected both in circulation and atherosclerotic lesions, although the structural and functional properties of circulating and plaque-resident ox-HDLs may be different. Indeed, site-specific modifications of ApoA-I have been identified in ox-HDLs present in plaque, but not in circulating ox-HDLs, resulting in distinct biological functions [73].

However, unlike HDLs isolated from healthy subjects, ox-HDLs obtained from patients with both stable coronary artery disease or acute coronary syndrome show reduced anti-inflammatory properties and lack the ability to induce endothelial repair. Indeed, these HDLs activate endothelial protein kinase C beta II (PKCβII) through interaction with LOX-1, leading to the inhibition of endothelial nitric oxide synthase (eNOS)-dependent nitric oxide (NO) production [74].

The detrimental effects of ox-HDLs are multiple. In addition to losing the antiatherogenic properties of HDLs, ox-HDLs induce proliferation and migration of smooth-muscle cells (SMCs), promote endothelial dysfunction and platelet activation, reduce migration of macrophages, and induce apoptosis [75,76,77,78,79]. Although the mechanisms underlying these effects are poorly understood, it has been shown that ox-HDL can bind LOX-1, and this interaction results in the harmful action on ECs [80]. In particular, it has been observed that ox-HDLs, but not HDLs, bind the LOX-1 receptor, inducing an increase in ROS production, and inducing the expression of LOX-1 in ECs by promoting its localization on the plasma membrane. Regarding the potential mechanisms involved, Perez et al. demonstrated the activation of NOX-2 and NF-ķB molecular pathways, suggesting a positive feedback mechanism in oxidative stress conditions [81].

In a complex environment such as an atherosclerotic lesion, various oxidative agents can oxidize HDLs. Indeed, HOCl-modified lipoproteins, which are present in human atherosclerotic plaque, can be generated by hypochlorous acid (HOCl) produced in vivo from the myeloperoxidase/H_2_O_2_/halide pathway, which is a potent oxidizing agent [82]. Consequently, HOCl-HDL can bind LOX-1, leading to endothelial internalization and degradation of modified lipoproteins [83].

Modified HDL can also derive from a point mutation in the human *APOA-I* gene, resulting in altered HDL-cholesterol levels and/or increased risk of cardiovascular disease [84,85,86]. It has been demonstrated that the generation of three natural mutations (ApoA-I) in HDLs alters the structure of the native lipoproteins, leading to an impairment of their functions. However, only one of these mutations (Apo-A-I[A164S]) was found to be an accurate predictor of an increased risk of myocardial infarction and mortality. One of the proposed mechanisms for the effect of this mutation is the activation of LOX-1 by apo-A-I[A164S] [87].

## 5. LOX-1 Receptor

The LOX-1 is a type II membrane protein with a molecular weight of approximately 50 kDa, belonging to the C-type lectin family. It was first described in 1997 when its role as the SR responsible for the up-taking of ox-LDLs by ECs was uncovered by Sawamura et al. [88]. However, LOX-1 is present also in other cell types, including SMCs, macrophages, lymphocytes, neutrophils, fibroblasts, dendritic cells, and cardiomyocytes [89]. In addition, it is present in atherosclerotic lesions and is involved in cell dysfunction and plaque formation [90,91]. Several in vivo studies have shown that, while basal expression of LOX-1 is very limited in physiological conditions, its levels are elevated in damaged endothelium during atherosclerosis [10,92,93], hypertension [94], and myocardial ischemia [95]. Furthermore, it is induced by several stimuli involved in the atherosclerotic process, including ox-LDLs, inflammatory cytokines, oxidative stress, vasoconstrictive peptides, and stress [96,97]. Moreover, the activation of LOX-1 modulates several pathological processes, especially proatherogenic pathways, resulting in endothelial dysfunction, SMC migration, monocyte recruitment, foam-cell formation, and platelet activation [98]. All this evidence suggests that LOX-1 may be an attractive therapeutic target to counteract atherosclerosis [99].

## 6. LOX-1 Gene

In humans, the LOX-1 protein is encoded by a single-copy gene known as ox-LDL receptor 1 (OLR1, OMIM No. 602601), which is localized on the short arm of chromosome 12, in the distal portion of the 12p13.2-p12.3 region. The OLR1 gene presents a structure similar to the natural killer (NK) cell receptor and is located in the NK gene complex, suggesting that both of these genes have derived from a common ancestor gene, and during evolution, LOX-1 has acquired a unique transcriptional profile [100].

The gene extends more than 7000 bp and comprehends six exons separated by five introns, with a length spanning approximately 15 kb [101]. Exons 1 to 5 are relatively small in length, ranging from 102 to 246 bp. Exon 6 is significantly longer, with a length of 1722 bp, while the inducible promoter and enhancer regions of this gene cover a length of approximately 2500 bp. The TATA and CAAT boxes of this gene are located in the proximal portion of the 5′ flanking region, respectively, at −29 bp and −99 bp [102].

Exon 1 encodes for the 5′-untranslated region (UTR) and the cytoplasmic portion of the receptor, exon 2 encodes for the remaining part of the cytoplasmic domain and the transmembrane domain, exon 3 encodes for the NECK domain, and exons from 4 to 6 encode for the 3′UTR and the C-terminal domain CTLD.

The 5′-flanking region has a functional promoter activity containing binding sites for transcription factors, such as the STAT family and NF-IL6, and some potential transcription factors binding sites, including the GATA-2 binding site, c-ets-1 binding element, 12-O-tetradecanoylphorbol 13-acetate-responsive element, and shear-stress-responsive elements, can contribute regulating the expression of LOX-1 in the endothelium [101]. Moreover, Metha et al. have identified that the region between nt-1494 and -1599 is necessary for the activation of the LOX-1 promoter induced by ox-LDLs [96].

Several studies have shown different polymorphisms in the ORL1 gene that were associated with coronary artery disease and acute myocardial infarction [103,104,105,106].

The combined effects of alternative splicing and single nucleotide polymorphism determine the production of different LOX-1 protein isoforms. Numerous factors can modulate the expression of specific LOX-1 isoforms, such as environmental factors, ILs, chromatin modulators, and transcription factors [107]. Alternative splicing of the ORL1 gene leads to the production of three *Lox-1* mRNA variants: transcript variant 1 (NM_002543), transcript variant 2 (NM_001172632), and transcript variant 3 (NM_001172633). The first transcript variant corresponds to the full-length OLR1, and it is characterized by the presence of all six exons, with an overall length of 2462 bp, which is responsible for the transcription of a complete LOX-1 isoform with an active ox-LDLs binding function. Transcript variant 2 is the shortest in length (950 bp), and it is characterized by the absence of exon 4, resulting in a shorter protein. Moreover, the C-terminal region of this protein lacks the ligand-binding and recognition domains. Transcript variant 3 is deprived of exon 5, and it encodes for a protein with a truncated CTLD, known as LOXIN [107]. The integrity of the CTLD domain is fundamental for ox-LDLs binding [108,109]; therefore, LOXIN shows the incapability to bind ox-LDLs. Also, the LOXIN can dimerize with the functional LOX-1 isoform, resulting in the inhibition of its activity [110].

## 7. LOX-1 Protein Structure

The functional isoform of LOX-1 is a homodimer consisting of four distinct domains: the N-terminal cytoplasmic domain, the transmembrane domain, the NECK domain, and the C-terminal domain CTLD, which is also called the C-type lectin-like domain.

In particular, the small intracellular N-terminal domain is linked to a hydrophobic transmembrane domain [111], and the extracellular region is constituted by the NECK domain, a coiled-coil structure of 80 amino acids, connected to the CTLD, a coiled structure of 130 amino acids. Antiparallel β-sheets flanked by α-helices compose the structure of this domain, which is further stabilized by three intrachain disulfide bridges. Notably, six conserved cysteine residues link CTLD monomers [112], forming an interchain disulfide bridge that favors the constitution of a central hydrophobic tunnel spanning the entire protein. Nonpolar amino acids cover the walls of this structure, determining the efficient transfer of lipids [111].

In LOX-1, ligand recognition is determined by the CTLD region [108]. The ability of CTLD to bind negatively charged ligands, such as ox-LDLs, is attributed to a positively charged amino acid terminal cluster (called the basic spine) [109]. CTLD domain deletion completely abolishes ox-LDLs’ binding activity, demonstrating this domain’s importance for LOX-1 function [108]. Additionally, ox-LDL binding is abolished by the deletion or sequential substitution of CTLD basic spine residues with alanine residues, indicating that this terminal chain is required for ligand binding [108,113]. It is noteworthy that point mutations replacing charged terminal residues with uncharged residues completely abrogate ox-LDLs binding activity [113]. Positively charged residues in CTLD are necessary for ox-LDLs binding. Indeed, as demonstrated by Falconi et al. using structural modeling of the CTLD domain, it has shown that the alteration of its conformation when arginine residues are replaced with alanine strongly affects the ox-LDL binding [114]. Overall, these findings suggest that CTLD binds a variety of negatively charged ligands, including ox-LDLs, through positively charged C-terminal residues.

The NECK domain is another important domain of LOX-1 for its activity. In its earliest identification, NECK has been described as an 80-residue coiled coil that is connected to transmembrane domains, while the C-terminal portion is connected to CTLD through an interchain disulfide bond [115]. NECK’s proximal third is structurally less stable than the remaining domains, which has been identified as the target of proteases responsible for releasing 34 kDa soluble forms (sLOX-1) into the bloodstream after LOX-1 juxta membrane cleavage between Arg88 and Gln89 residues [116]. There is evidence that IL-18 plays a role in NECK domain cleavage and sLOX-1 release in a disintegrin and metalloproteinase domain-containing protein-10 (ADAM10)-dependent manner [117]. Furthermore, mutation of the C140 residue in CTLD abrogates the stability of the C-terminal NECK domain, indicating that CTLD integrity is required to maintain coiled-coil NECK structures [115]. Deletion of the NECK domain does not impair ox-LDL binding affinity, suggesting that NECK is not involved in ligand-binding activity [108]. Altogether, this evidence indicates that the NECK domain possesses a proximal N-terminal portion that is involved in the secretion of sLOX-1, and a distal portion that interfaces with the CTLD domain to increase its stability.

## 8. LOX-1 Ligands

Although LOX-1 was initially discovered as a receptor for ox-LDLs, subsequent studies have demonstrated that it recognizes and binds with high affinity also other classes of compounds, including modified lipoproteins such as acetyl-LDL, hypochlorite-modified high-density lipoproteins, carbamylated-LDL, remnant-like lipoprotein particle, and electronegative LDL, but also erythrocytes, leukocytes, activated platelets, aged and apoptotic cells, bacterial products, advanced glycation end products, free fatty acids, and C-reactive protein [118,119,120,121,122].

LOX-1 exists on the cell surface as a covalent homodimer resulting from the formation of an intermolecular disulfide bond of Cys140 in the NECK domain. The dimer is the minimal structural unit of LOX-1. By crosslinking studies, it has been shown that these homodimers can form high-order oligomeric complexes, such as tetramers, and hexamers, that can contribute to the recognition and internalization of the ligands [112]. Receptor oligomerization is a process by which cell surface receptors increase their capability to bind ligands and depend on the density of the receptors on the plasma membrane. It is crucial for the ligand-binding function; indeed, it is proposed as a potential mechanism for the high-affinity binding of LOX-1 with the ligands [123].

## 9. LOX-1 and ROS Generation

Several chemical and biological stimuli induce ROS generation in vascular tissue as well as in circulating cells [121,124]. ROSs are important intracellular messengers able to affect numerous signaling pathways including LOX-1 expression [125]. In addition, LOX-1 activation per se can induce ROS generation, suggesting a positive feedback mechanism between LOX-1 and ROS [126].

It is known that the binding of ox-LDLs to LOX-1 results in a marked increase in intracellular ROS generation, including O_2_^−^, H_2_O_2_, peroxynitrite, nitric oxide (NO), and hydroxyl radicals [127]. Among them, O_2_^−^ is responsible for the inactivation of NO by a chemical reaction that leads to the generation of peroxynitrite [128]. In addition, the O_2_^−^ generated induces LOX-1 expression, creating a vicious circle with a further increase in ROS generation. This interaction may result in a benefit, especially in a condition of low levels of oxidative stress; indeed low levels of ox-LDLs induce capillary tube formation through the activation of NADPH oxidase LOX-1-mediated [129].

However, if the ROS generation becomes excessive, the ROS-LOX-1 axis results in cell damage.

The activation of the NADPH oxidase complex is a key step in the generation of ROS induced by LOX-1 stimulation, and the increased ROS generated from NADPH oxidase contributes to the onset and progression of atherosclerosis through the activation of several molecular pathways [130]. So, the activation of NADPH oxidase triggers a redox signal involving p38 MAPK and the transcription factor nuclear factor-kB (NF-ķB), resulting in an increased expression of pro-inflammatory mediators and adhesion molecules [131].

The interplay between LOX-1 and NADPH oxidase has been shown also in an animal model of atherosclerosis. The upregulation of the four subunits of NADPH (p47phox, p22phox, gp91phox, and Nox-4 subunits) detected in LDLR^−/−^ mice compared to wild-type mice was indeed reduced in LOX-1^−/−^/LDLR^−/−^ mice, with a consequent decrease of the redox-sensitive signals [132]. Furthermore, the deletion of LOX-1 reduced the increase of both the p22phox and p47phox subunits during ischemia–reperfusion injury in LOX-1^−/−^ mice [133].

Although additional studies are required to better clarify the role of NADPH oxidase in modulating LOX-1 expression, these data highlight the important link between LOX-1 and NADPH oxidase.

The production of ROS induced by the binding of ox-LDL to LOX-1 activates pathways involved in important cellular functions, such as phagocytosis [134] and apoptosis [135], functions that are mediated by the activation of the redox-sensitive transcription factor NF-kB [136] that contributes to plaque destabilization. In this scenario, it has been demonstrated that the anti-LOX-1 antibody significantly reduced the ROS production, NF-ķB activation, and apoptosis induced by the uptake of ox-LDL [125,137].

## 10. LOX-1 Signalling Pathways and Their Targets

The various biological effects induced by the activation of LOX-1 have been mainly studied using ox-LDLs as ligands [138], and although several transcription factors are involved in the downstream pathways, most of the studies focused on the important role of NF-kB.

Furthermore, in the 5′-regulatory regions of the LOX-1 gene, an NF-kB binding element [101] is present. Thus, the binding of ox-LDLs to LOX-1 activates NF-kB, which, in turn, binds to the shear-stress-responsive element binding site GAGACC in the 5′-regulatory regions, inducing the expression of LOX-1. In addition, the binding of ox-LDLs to LOX-1 leads to the activation of inflammatory pathways with the expression of adhesion molecules and the synthesis of proinflammatory cytokines, giving rise to a vicious cycle of ox-LDLs uptake by LOX-1, increased ROS generation, and increased LOX-1 expression [135].

The promoter region of LOX-1 also contains the recognition sequences for the activator protein 1 (AP1) transcription factor. Therefore, the binding of LOX-1 ligand to the receptor triggers a plethora of signaling pathways involved in several pathological conditions, including oxidative stress, inflammation, apoptosis, lipid metabolism, and angiogenesis, which collectively promote atherosclerotic plaque formation and progression [98].

The activation of LOX-1 may activate intracellular protein kinases, such as ERK1 and ERK2, and p38MAPK kinases [138,139,140,141,142]. As a result, it has been shown that an increased expression of monocyte chemoattractant protein-1 (MCP-1) and adhesion molecules resulted in the enhanced adhesion of monocytes to the endothelium. Moreover, LOX-1 activation inhibits the phosphorylation and activity of Akt kinase, which is associated with a reduction of eNOS activity [142].

After ox-LDLs activation, a LOX-1-membrane type 1 metalloproteinase (MT1-MMP) axis has been shown to play a crucial role in the activation of the signaling pathways mediated by RhoA and Rac1 GTPases [143]. RhoA activation inhibits the eNOS synthesis, while that of Rac1 increases the NADPH oxidase activity, leading to ROS production and oxidative stress induction. Inhibiting LOX-1 or MT1-MMP has been shown to prevent eNOS downregulation induced by RhoA activation, NADPH Rac-1-mediated activity, and ROS generation [143].

The dimerization of LOX-1 plays a critical role in the subsequent MAP kinase activation. Indeed, the heterodimerization with a dominant-negative receptor carrying mutations in the extracellular domain significantly reduced the signaling response to ox-LDLs [126] without affecting the binding of ox-LDLs to the receptor complex. The dimerization process is essential for the recruitment of cytosolic adaptor proteins, with ARHGEF1 (rho guanine nucleotide exchange factor 1) and ROCK2 (rho associated coiled-coil containing protein kinase 2) identified as potential interaction partners [144]. While ARHGEF1 is constitutively bound to LOX-1, ROCK2 is detected in receptor complexes only after ox-LDLs stimulation. The inhibition of ROCK2 results in the lack of NF-kB activation and its downstream effects, such as cytokine production, demonstrating the critical role of ROCK2 in LOX-1 signaling [144].

LOX-1 downstream targets have been more extensively characterized than their signaling mechanism. LOX-1 activation reduces the production of NO by the endothelium, which promotes vasodilation and reduces platelet activation [143,145]. Further, LOX-1 activation significantly promotes the expression of different target genes. Indeed, in HAECT (human aortic endothelial cells transformed-1) cells, the activation of LOX-1 by ox-LDLs significantly promotes the expression of different target genes related to inflammation, such as cell adhesion and upregulation of cytokines including IL-8, CXCL2 (C-X-C Motif Chemokine Ligand 2), CXCL3 (C-X-C Motif Chemokine Ligand 3) and CSF3 (Colony Stimulating Factor 3) [135]. Furthermore, the overexpression of the LOX-1 receptor in fibroblasts increases the expression of ICAM-1 (intercellular adhesion molecule 1) and VCAM-1 (vascular cell adhesion protein 1), mediating the firm adhesion of leukocytes to ECs as detected in different inflammatory diseases [146].

## 11. LOX-1 Inhibitors: Natural and Synthetic Compounds

The evidence provided above suggests that LOX-1 could be an important therapeutic target for atherosclerosis and associated diseases [147]. Below is an overview of the different compounds, natural or synthetic, that can modulate LOX-1 expression.

Many natural bioactive compounds can modulate LOX-1 expression and its downstream signaling pathways, exerting anti-atherosclerotic effects [148]. One of these compounds is curcumin, a natural polyphenolic compound extracted from the rhizomes of Curcuma longa L. It possesses antioxidant and anti-atherosclerotic properties, as confirmed by multiple clinical studies showing that treatment with curcumin ameliorated dyslipidemia, increased NO levels, and improved endothelial function [149,150,151,152]. Several molecular mechanisms are involved in the anti-atherosclerotic effects of curcumin, including a reduction in PPAR-γ and CD36 expression, as detected in macrophages; an increase of cholesterol efflux through ABCA1; and the blockage of superoxide production shown in cultured porcine artery rings [153]. In vitro studies in HUVECs have also shown that curcumin inhibits LOX-1 expression in a concentration-dependent manner [154], suggesting that the anti-atherosclerotic properties of this natural compound are due, at least in part, to the inhibition of LOX-1.

Tanshinone II-A is a pharmacologically active compound extracted from the rhizome of the Chinese herb Salvia miltiorrhiza Bunge, used in traditional Chinese medicine for the prevention and management of cardiovascular diseases. In the murine macrophage cell line RAW264.7, it has been shown to inhibit the LOX-1 expression induced by ox-LDLs and ROS production. The molecular mechanism involved includes the suppression of the nuclear translocation of the NF-kB p65 subunit and the phosphorylation of IkB-α induced by the internalization of ox-LDLs via LOX-1 [155]. The anti-atherosclerotic property of Tanshinone II-A has been demonstrated in an in vivo study in ApoE^−/−^ mice, in which it has been shown that the oral administration of this compound reduces LOX-1 expression with a marked decrease in atherosclerotic lesion size [155]. In addition, in this animal model, transhinone II-A markedly reduced NLRP3 inflammasome activation [156].

Many experimental and epidemiological studies have demonstrated that dietary polyphenols show an anti-atherosclerotic effect due to an anti-inflammatory and anti-oxidant action [157]. Accordingly, several studies have demonstrated the beneficial effects of resveratrol in the treatment of cardiovascular diseases [158]. Resveratrol is mainly present in grapes and red wine, and resveratrol supplementation prevents oxidative stress by modulating several proteins involved in the oxidation processes. Moreover, it lowers blood pressure, improves endothelial function, increases NO production, inhibits platelet aggregation, and the synthesis of inflammatory prostaglandins [157]. Although the mechanisms behind these effects are not fully understood, one of the metabolic pathways affected by resveratrol is the suppression of LOX-1 expression induced by ox-LDLs [159].

Furthermore, research has also shown that bergamot-derived polyphenolic fraction (BPF) can affect LOX-1. In particular, it has been found that BPF can decrease LOX-1 expression in polymorphonuclear leukocytes of hyperlipidemic patients, as demonstrated by Gliozzi et al. [160]. In addition, the combination of BPF polyphenolic fraction with rosuvastatin has been shown to enhance rosuvastatin’s antioxidant activity and normalize the serum lipemic profile of hyperlipidemic patients. This combination also leads to an increase in the expression and phosphorylation of protein kinase B, which results in vasoprotective and anti-proliferative effects, contributing to reduced vascular damage [160].

Feng et al. conducted a study wherein they discovered that Ginkolide B from Ginkgo Biloba has protective effects in endothelial dysfunction. In vitro experiments, performed on HUVECs and RAW267.7 macrophages, have shown that the expression of LOX-1 was inhibited after treatment with Ginkgolide B, resulting in the reduction of lipid and cholesterol accumulation in the treated cells [161].

To identify molecules that can block LOX-1, a cellular high-throughput screening assay was conducted by Schnapp et al. [162]. More than one million small compounds were tested for their capacity to bind LOX-1 and block the AlexaFluor594 (AF594)-labeled human ox-LDL uptake into a CHO-K1 cell line expressing the human LOX-1. The resulting hits underwent a counter screening, similar to the first assay, but using a counter target, the scavenger SR-BI. This receptor plays a key role in the HDL-C uptake mainly in the liver and has a structural homology to human LOX-1. All the small molecules that blocked both receptors via an unspecific mechanism were excluded from the analysis. Among the molecules that showed selective inhibition of LOX-1, the compound BI-0115 was the most active compound, with a marked capacity to inhibit the ox-LDL internalization without activity on the SR-BI receptor. The compound BI-0115 was tested on various pharmacological targets, including nuclear receptors, ion channels, G protein-coupled receptors, drug transporters, kinases, and non-kinase enzymes, to ensure that it does not have any off-target actions. The tests showed that BI-0115 did not affect any of these targets. Although the compound has low solubility, it is still sufficient to produce a saturable dose response in both cellular and biophysical assays. Based on these findings, BI-0115 is being proposed as a tool for in vitro studies. Recently, in a rat thromboembolic stroke model, it has been demonstrated that LOX-1 inhibition with BI-0115 markedly improved several neurological functions and the global outcomes following ischemic stroke and recombinant tissue-type plasminogen activator (rt-PA) therapy. In detail, BI-0115 significantly reduced the edema formation and the infarcted area and improved neurological function [163]. In addition, a marked increase of the barrier integrity was observed after treatment with BI-0115 of brain microvascular endothelial cells exposed to hypoxia plus glucose deprivation and reperfusion [163]. However, further studies are needed to confirm whether it can be used as a selective inhibitor of LOX-1 [162].

Statins are widely used in the treatment of cardiovascular disease due to their cholesterol-lowering effects. The mechanism of action involves the inhibition of the 3-hydroxy-3-methylglutaryl coenzyme A (HMG-CoA) reductase, which is the major rate-limiting enzyme in cholesterol biosynthesis. Moreover, statins may also affect the function of LOX-1 by other mechanisms. LOX-1 is mainly localized in caveolae and lipid rafts in the plasma membranes, and the levels of cholesterol in the membrane modulate its activity. Accordingly, the chronic exposure of primary ECs to lovastatin by a reduction of the cholesterol levels in the membrane led to a disruption of the lipid raft and spatial disorganization of LOX-1, with a consequent significant reduction of ox-LDL uptake via this receptor. Lovastatin also prevented the ox-LDL-induced apoptotic phenotype, suggesting that the disruption of LOX-1 by statins protects the vascular endothelium and counteracts the effects of ox-LDL [164].

It is known that statins exert many anti-atherosclerotic effects that are not mediated by cholesterol. Among these pleiotropic effects, Biocca et al. have provided evidence that statins, in addition to their indirect action on LOX-1 activity dependent on the reduction of the intracellular levels of cholesterol, can inhibit the binding of ox-LDLs to LOX-1 by direct interaction with the C-type lectin-like recognition domain of LOX-1 [165].

Another class of drugs affecting LOX-1 activity is the angiotensin-converting enzyme (ACE) inhibitors and AT1 receptor blockers, drugs that are commonly used in the treatment of primary hypertension [166]. In particular, it has been shown that the AT1 blocker Losartan reduces the upregulation of LOX-1 and the subsequent internalization of ox-LDLs induced by Angiotensin II [167,168]. Similarly, the ACE inhibitor Quinapril decreases the levels of LOX-1, p22phox, p47phox, and gp91phox in the left ventricle of Dahl salt-sensitive hypertensive rats fed a high-salt diet, suggesting a protective role due to the inhibition of oxidative stress mediated by LOX-1 [169].

The use of anti-LOX-1 antibodies has been shown to have numerous positive effects on atherosclerotic disease. For instance, in vitro studies have shown that anti-LOX-1 monoclonal antibodies reduce the formation of ROS induced by ox-LDL [125] and increase the production of intracellular NO in HUVEC [126].

In addition, an in vivo study has demonstrated that an anti-LOX-1 monoclonal antibody protects against myocardial ischemia–reperfusion injury in a murine model. In particular, this study has shown that the treatment of Sprague–Dawley rats with the LOX-1 blocking antibody prevented the ischemia–reperfusion-induced upregulation of LOX-1 and significantly reduced MMP-1 and adhesion molecule expression. It also reduced leukocyte recruitment in the damaged area, leading to a significant reduction in myocardial infarct size [170]. In another study by Nakano and colleagues, it was found that treatment with an anti-LOX-1 antibody reduced lipid deposits in the mesenteric arteries of stroke-prone spontaneously hypertensive rats [171]. Furthermore, the treatment with the neutralizing antibody LOX-1 prevented coronary endothelial dysfunction, reduced superoxide production, and the expression of LOX-1 in ApoE^−/−^ mice. It also increased eNOS expression and restored coronary arteriolar dilatation in ApoE^−/−^ mice without affecting these parameters in wild-type control mice [172].

Below a summarized table of LOX-1 inhibitors and their role in cardiovascular diseases (Table 1). 

## 12. Regulation of LOX-1 Expression: In Vitro and In Vivo Studies

Numerous studies have been conducted to comprehend the molecular mechanisms that regulate LOX-1 activity and explore the correlation between LOX-1 and atherosclerosis, owing to the crucial role of LOX-1 in the pathophysiology of cardiovascular diseases. These studies have led to the development of various strategies aimed at modulating or inhibiting LOX-1 gene and/or protein expression.

### 12.1. RNA Silencing

One of the methods used to regulate gene expression is RNA silencing. Understanding the specific molecular mechanisms underlying the involvement of LOX-1 in atherosclerosis is essential for developing targeted approaches, and by cell silencing techniques, it has been possible to better understand the functions of LOX-1 [98]. This process involves the suppression of the gene regulatory mechanism, either suppressing transcription or activating RNA degradation. The mechanism involves (i) cleavage of the double-stranded RNA by an RNase III-like activity into small interfering RNA (siRNA), (ii) loading of one strand of siRNA onto an Argonaute protein possessing nucleolytic activity, and (iii) guidance of siRNA to the cognate RNA and its slicing with subsequent repression or degradation [173].

RNA silencing can be performed with both a sense and an antisense transgene, which act by a similar mechanism [174]. The use of antisense oligonucleotides to inhibit the expression of LOX-1 mRNA was first reported by Li and Mehta [139]. They found that this approach effectively suppressed LOX-1 upregulation caused by ox-LDLs and prevented MCP-1 and oxLDLs-induced LOX-1 monocyte adhesion to human coronary artery endothelial cells (HCAECs). Additionally, the authors also showed that specific antisense to LOX-1, as well as the chemical inhibitors polyinosinic acid and carrageenan, reduced the proapoptotic effect of ox-LDLs. In this scenario, it has been demonstrated that the inhibition of NF-kB-mediated signal transduction is a mechanism involved in LOX-1-mediated apoptosis [175].

Research studies investigating the role of LOX-1 in activating macrophages have shown that, when the LOX-1 gene is silenced through siRNA transfection, there is a reduction in ROS production, autophagy, mitochondrial DNA damage, and decreased expression of the NLRP3 inflammasome in THP-1 and primary peritoneal macrophages of mice. This confirms the key role of LOX-1 in the inflammatory molecular pathway [176]. Similarly, the silencing of the LOX-1 gene in mouse RAW264.7 macrophages using siRNA resulted in an attenuation of the oxidative stress induced by ox-LDLs.

According to a study by Ishino et al., the expression of LOX-1 is related also to atherosclerotic plaque instability and fibrous cap rupture in hyperlipidemic rabbits [177]. Similarly, in a mouse model (ApoE^−/−^) of high-fat diet-induced atherosclerosis, the delivery of adenovirus LOX-1 siRNA was found to increase the fibrous cap thickness and reduce the number of macrophages recruited in the atherosclerotic lesion. This suggests that LOX-1 silencing may protect against atherosclerosis by preventing oxidative stress, reducing macrophage accumulation, and downregulating NOX enzymes [178]. In addition, silencing the LOX-1 gene in mouse RAW264.7 macrophages using siRNA resulted in an attenuation of the oxidative stress induced by ox-LDLs. Overall, these findings indicate that antisense oligonucleotides, short single-strand DNA, or RNA molecules that target LOX-1 can be a valuable strategy for reducing LOX-1 expression and counteracting the development and progression of atherosclerotic plaque.

### 12.2. miRNA

MicroRNAs (miRNAs) are small, single-stranded non-coding RNAs that are approximately 18–24 bp nucleotides long. They regulate gene expression at the post-transcriptional level by binding to the 3′ untranslated region (UTR) of target mRNA sequences. This binding blocks the mRNA translation and leads to its degradation [179]. It is estimated that miRNAs regulate more than 60% of protein-coding genes, and a single miRNA can interact and modulate several targets involved in different or the same molecular pathways [180]. Several studies have highlighted the significant roles that miRNAs play in cardiovascular diseases, including atherosclerosis. For instance, miRNAs can decrease lipid synthesis and preserve the vascular endothelium [181,182], regulate lipoprotein homeostasis, control cholesterol and fatty-acid metabolism [183,184,185], modulate macrophage activation, and inhibit their transformation into foam cells [186].

It has been recently discovered in cellular and animal models that miRNAs can modulate the expression of LOX-1 mRNA, leading to a reduction in atherosclerosis [187]. In this regard, it has been demonstrated that the 3′ UTR region of LOX-1 mRNA contains a binding site for the let-7g, which is an miRNA involved in maintaining endothelial function and vascular homeostasis, as well as regulating the proliferation and apoptosis of ECs and vascular SMCs [188]. The anti-atherosclerotic activity of let-7g depends, at least in part, on its direct inhibition of LOX-1 [189]. In an in vivo study conducted on ApoE knock-out mice fed a high-fat diet for 6 weeks, an increase of circulating ox-LDL and LOX-1 gene expression was observed, which was associated with a decreased expression of miRNA let-7g [187].

It has been demonstrated that LOX-1 plays a role in atherosclerosis, even in a cellular model of transfected human aortic smooth-muscle cells (ASMC). Overexpression of LOX-1 in this model resulted in cell migration and proliferation. However, these effects were nullified when co-transfected with miRNA let-7g [189]. Additionally, administering miRNA let-7g in ApoE knockout mice that were fed a high-fat diet for 12 weeks led to a substantial reduction in LOX-1 mRNA and protein levels, and also decreased neointimal hyperplasia compared to scramble-treated aortas. Morphometric analyses revealed significant improvements in key atherosclerosis severity parameters, such as the lumen area and intima area, after miR-let-7g mimic delivery [189].

Dai et al. used bioinformatic prediction to identify the direct target of miR-98 as the 3′-UTR regions of the LOX-1 gene. They found that treatment of macrophages with ox-LDL resulted in an increase in LOX-1 expression and a decrease in miR-98 levels [190]. The authors also investigated the relationship between miR-98 and LOX-1 in foam-cell formation. Their results showed that the transfection of macrophages with an miR-98 mimic led to a decrease in LOX-1 mRNA and protein levels, while inhibition of this small RNA was associated with an increase in both LOX-1 mRNA and protein levels. Interestingly, the reduction of miR-98 and the concomitant increase of LOX-1 were associated with a transformation of macrophages into foam cells, suggesting that LOX-1 can be a mediator also in miR-98-regulated atherogenesis [190]. In addition, miR-98 has been found to exhibit protective properties against the ox-LDL-induced apoptosis that is mediated by LOX-1 activation in HUVECs. This is achieved through the reduction of the ox-LDL receptor expression and the preservation of cell proliferation and epithelial functions. These findings suggest that LOX-1 may have a significant role in the molecular mechanisms that regulate endothelial injury that contribute to the pathogenesis of atherosclerosis through the regulation of multiple pathways [191].

Based on the data available in the literature, LOX-1 has been identified as a potential new contributor to the atherosclerotic process. It has been found that LOX-1 expression can be modulated by various miRNAs, such as miR590-5p and miR-9. By targeting LOX-1 and suppressing redox-sensitive signals, miR590-5p reduces angiogenesis in HUVEC. On the other hand, miR-9 negatively regulates the LOX-1-mediated p38 MAPK pathway, thereby reducing the formation of vulnerable atherosclerotic plaque in ACS mice [192,193].

### 12.3. Modulation of LOX-1 in Transgenic Animal Models

The impact of reducing LOX-1 was studied in multiple animal models, including transgenic ones. Mehta et al. found that this approach significantly reduces the formation of atherosclerosis not only in LOX-1-deficient mice that were fed with a high-fat diet compared to regular mice but also in LDLR-KO/LOX-1-KO compared to LDLR-KO. Additionally, by deleting LOX-1 in LDLR-KO mice, there was a decrease in cell migration and the blocking of macrophage trafficking in the aorta, which highlights the role of LOX-1 in the progression of atherosclerotic plaque. Furthermore, double-knock-out animals showed a reduction in markers of oxidative stress and inflammation, such as superoxide dismutase (SOD) and IL-10, and preservation of endothelial integrity [194,195].

The role of LOX-1 in promoting atherosclerosis was also investigated in mice that were genetically modified to have endothelial-specific LOX-1 overexpression with an ApoE deficiency background. These mice exhibited an increase in lipid deposition and inflammation in the aorta, along with increased endothelial dysfunction and the formation of atherosclerotic plaque [196]. Of note, as a marked increase of LOX-1 was also detected in human atherosclerotic plaque [197], its endothelial-specific inhibition could represent a potential target for the prevention and treatment of atherosclerosis.

Furthermore, LOX-1 is also known to play a crucial role in myocardial infarction and associated damage due to ischemia. In this scenario, Lu et al. [198] have demonstrated that LOX-1-deficient mice had a higher survival rate than wild-type C57BL/6 mice over three weeks follow up from total ligation of the left coronary artery. This finding provides evidence that LOX-1 expression plays a crucial role in the cardiac remodeling process that occurs after chronic myocardial ischemia. Additionally, it has been observed that the removal of LOX-1 preserves the systolic thickness of the left ventricle anterior wall and ejection fraction in animals with chronic ischemia compared to corresponding sham ischemia animals. Furthermore, the deletion of LOX-1 also results in a lower deterioration of overall cardiac functions, collagen deposition reduction, and reduction in myocardial oxidative stress. Moreover, it has been demonstrated by Hu et al. that LOX-1 deficiency protects against ischemia–reperfusion damage [199]. LOX-1 KO mice, exposed for 60 min to coronary artery occlusion followed by 60 min of reperfusion, showed a reduction in the extent of infarction, a decrease in inflammatory cell infiltration, and reduced lipid peroxidation. Additionally, the reduction in the necrotic area was associated with the preservation of cardiac function.

Overall, these findings suggest that LOX-1 abrogation might be of help in treating both acute and long-term ischemia [198].

## 13. LOX-1 in Atherosclerosis

Atherosclerosis is a chronic inflammatory disease with a complex pathophysiology. The onset of this disorder is triggered by the activation of pro-inflammatory signaling pathways, the expression of cytokines and chemokines, and an increase in oxidative stress [200]. In a condition of oxidative stress, LDLs undergo modifications and become ox-LDLs, which are potent atherosclerotic mediators [124]. Ox-LDLs activate the inflammatory and immune response, induce the adhesion of monocytes to ECs, and promote the release of growth factors that favor the development and progression of atherosclerotic plaque. Many of the effects induced by ox-LDLs are mediated by the activation of the receptor LOX-1, including endothelial dysfunction, monocyte adhesion, foam-cell transformation, smooth-muscle-cell proliferation, and migration [133,201,202], as described above.

The interaction between OxLDLs and LOX-1 causes an increase in intracellular ROS generation, including O_2_^−^, H_2_O_2_, peroxynitrite (ONOO^−^), NO, and hydroxyl radicals (OH^−^) [203]. In particular, O_2_^−^ is involved in a chemical reaction that leads to the inactivation of NO and the formation of cytotoxic ONOO^−^ [203]. This reaction also upregulates LOX-1 expression, resulting in further ROS generation [137].

In the microenvironment of the atherosclerotic plaque, ROSs are generated during the initial steps of its formation, together with the cytokines and chemokines released by T-lymphocytes, mast cells, and foam cells recruited in the lesion area. Importantly, ROS stimulates SMC migration and collagen deposition, contributing to affecting the composition of the atherosclerotic plaque. They also induce the release of metalloproteinases that degrade the fibrous wall of the plaque cap, inducing its rupture, and the expression of SRs on the SMC surface, including LOX-1, facilitating the uptake of ox-LDLs and guiding them to become foam cells [204].

**Figure 1:**
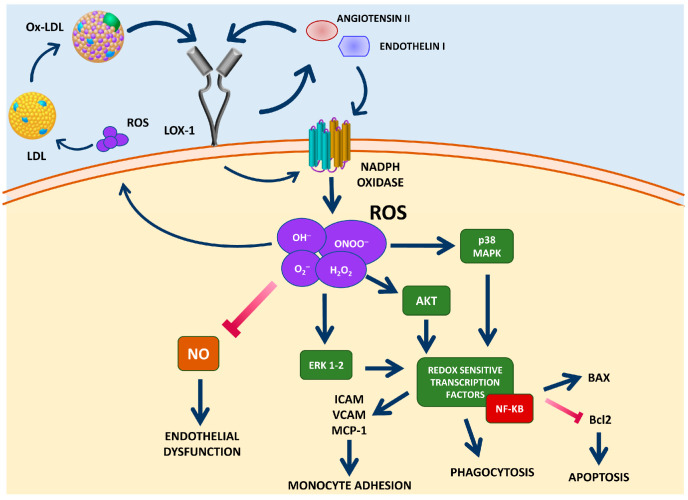
LOX-1 regulation, intracellular ROS production, and downstream effects. LOX-1 binds to circulating ox-LDLs or other ligands, such as Angiotensin II and Endothelin-1. This process activates NADPH oxidase, which causes the production of intracellular ROS, triggering different cascades, including p38-MAPK, ERK1-2, and AKT. The activation of these molecular pathways causes the induction of redox-sensitive transcription factors like NF-kB, leading to monocyte adhesion, phagocytosis, apoptosis, and endothelial dysfunction. In the circulation, released ROS can oxidize circulating LDL, resulting in the formation of ox-LDL, which can then bind and activate LOX-1, thereby increasing the intensity of this vicious cycle. Abbreviations: ox-LDL, oxidized LDL; LOX-1, lectin-like oxidized low-density lipoprotein receptor-1; ROS, reactive oxygen species; ICAM, intracellular adhesion molecule; VCAM, vascular cell adhesion molecule; NO, nitric oxide.

In summary, as represented in Figure 1, a vicious cycle of LOX-1, ox-LDLs, and ROS is established. This cycle involves increased levels of Ang II and ET-1, enhanced oxidative stress by higher ROS species formation, and Nox activity that leads to increased oxidation of LDL and further enhanced oxidative stress in response to ox-LDLs. Overall, this process favors endothelial dysfunction and atherosclerosis progression in the vessel wall [205,206].

## 14. Role of LOX-1 in Endothelial Dysfunction

Ox-LDLs are mainly internalized by LOX-1 in ECs, contributing to the development of endothelial dysfunction through different mechanisms [88]. Ox-LDLs and other atherogenic stimuli, such as proinflammatory cytokines, can induce LOX-1 expression, which is generally low in normal conditions [135], increasing both mRNA and protein expression in a dose-dependent manner [146]. The treatment of cells with an antisense LOX-1 mRNA significantly suppresses ox-LDL-induced upregulation of LOX-1, which confirms that ox-LDLs induce the expression of LOX-1 through interaction with its receptor [175]. Furthermore, ox-LDLs increase the expression of MCP-1, a chemotactic protein for circulating monocytes. This results in a significant increase in their adhesion to ECs, effects that can be suppressed by treating ECs with antisense LOX-1 mRNA [139]. Ox-LDL-mediated LOX-1 activation also determines the upregulation of endothelial adhesion molecules, such as E-selectin, P-selectin, VCAM-1, and ICAM-1, further contributing to leucocyte recruitment and adhesion. The treatment of ECs with antisense LOX-1 mRNA completely abolished these effects [207]. Moreover, it has been shown that the increased expression of CD40/CD40L in human coronary artery endothelial cells (HCAECs) induced by ox-LDLs was reduced after incubation with a LOX-1 blocking antibody and resulted in an inhibition of this proatherogenic pathways and ECs activation [208].

In vitro studies have shown that high levels of ox-LDLs can lead to cell death. Indeed, ox-LDLs can induce both necrosis and apoptosis, with the latter involving multiple pathways, such as ROS production, caspase and protein kinase activation, altered calcium homeostasis, and changes in proapoptotic/antiapoptotic gene expression [209]. The apoptosis of ECs results in an increased vascular permeability to cells and lipids, proliferation of smooth-muscle cells, and activation of the coagulation cascade, all of which contribute to the development of atherosclerosis. LOX-1 activation mediates the apoptotic effect of ox-LDLs that can be reduced by inhibiting LOX-1 using antisense mRNA or chemical inhibitors [175]. Moreover, apoptosis induced by ox-LDLs is mediated by activation of NF-kB, and the antisense mRNA of LOX-1 significantly inhibited ox-LDL-induced NF-kB activation [175].

Ox-LDLs bind to ECs and activate caspase-9 and caspase-3, as well as mitochondrial caspase activators, while simultaneously reducing B-cell lymphoma 2 (Bcl-2) and c-IAP-1 (cellular inhibitor of apoptosis protein 1), leading to the downregulation of antiapoptotic signaling and activation of proapoptotic pathways [210]. These effects are significantly reduced when cells are treated with antisense LOX-1 mRNA before ox-LDL exposure, supporting the observation that ox-LDLs modulate the expression and activity of relevant apoptosis players through the LOX-1 receptor [210]. Ox-LDL-induced apoptosis involves Fas activation, a death receptor that triggers apoptosis when it binds to its ligand FasL. In this regard, it has been shown that ox-LDLs increase the expression of both Fas and FasL in vascular cells inducing apoptosis. Accordingly, FasL-neutralizing antibodies reduce ox-LDL-induced apoptosis [209]. LOX-1 activation likely explains these effects since treatment with neutralizing LOX-1 antibody inhibits ox-LDL-induced activation of Fas-mediated apoptosis in ECs and Fas expression [211].

## 15. Role of LOX-1 in Smooth-Muscle-Cells Proliferation and Apoptosis

Smooth-muscle cells express LOX-1, which can be upregulated by stimuli like ox-LDLs and angiotensin II (Ang II) [187,212,213]. It has been shown that higher levels of ox-LDLs induce apoptosis of vascular smooth-muscle cells by LOX-1 upregulation, an effect that is counteracted by the treatment with anti-LOX-1 antibodies [214]. Ox-LDLs can also promote the expression of the proapoptotic Bcl-2-associated X protein (Bax) and suppress the antiapoptotic Bcl-2 [214]. Interestingly, LOX-1 has been observed to colocalize with Bax in human atherosclerotic plaques, especially in the rupture-prone shoulder region, indicating its role in plaque destabilization [214].

LOX-1 also plays a pivotal role in the transformation of smooth-muscle cells into foam cells. In this scenario, it has been demonstrated that treatment with anti-LOX-1 antibodies markedly inhibited the ox-LDL uptake induced by lysophosphatidylcholine (LPC) [215]. In accordance, the treatment with anti-LOX-1 antibodies significantly reduces the ox-LDL uptake and the lipid droplet accumulation in smooth-muscle-like cells, thus preventing the transdifferentiation into foam cells [216].

## 16. Role of LOX-1 in Macrophage and Foam-Cell Formation

Monocytes do not express appreciable levels of LOX-1. However, during their differentiation into macrophages, its expression increases [217]. Macrophages can take in ox-LDL and become lipid-rich foam cells through three different mechanisms, including LDLR, receptor-independent fluid-phase endocytosis, and scavenger receptors, such as SR-A, SR-B, CD68, CD36, and LOX-1. Under normal conditions, macrophages express low levels of LOX-1, and the contribution to the uptake is minimal (about 5%). However, several stimuli, such as ox-LDLs, LPC, inflammatory cytokines, and high-glucose levels, can induce its expression [135]. In an atherosclerotic environment, LOX-1 internalizes ox-LDLs by up to 40% [218]. Indeed, it has been shown that macrophages stimulated with LPC show a marked increase of LOX-1 expression with a concomitant increase in ox-LDL internalization and degradation, effects that were not observed in LOX-1-deficient cells [218]. As proinflammatory cytokines induce LOX-1 expression and inhibit other SRs, including SR-AI/II and CD36, it has been proposed that, in the atherosclerotic microenvironment, where the levels of these cytokines are high, the role of LOX-1 in macrophage ox-LDLs uptake may become prominent. These results highlight the relevance of LOX-1 in macrophage lipid accumulation and suggest its role in foam-cell generation and atherogenesis [218].

## 17. Role of LOX-1 in Platelets Activation

In contrast to CD36, a member of the SRs family that is constitutively expressed in resting platelets, LOX-1 is only present on the platelet surface after activation [219]. Accordingly, it has been demonstrated that, in comparison with resting platelets, activated platelets internalize substantial amounts of ox-LDLs, which contribute to their activation [220]. This leads to platelet aggregation and the release of serotonin [221]. Furthermore, ox-LDLs-activated platelets show an increased ability to adhere to ECs, induce an inflammatory response, and accumulate at the site of vascular lesions. In addition, ox-LDL-positive platelets can also contribute to inflammation in vascular tissues by inducing adhesion molecule expression on EC surfaces, reducing their regeneration and promoting foam-cell formation [220]. As a result of endothelial dysfunction, ECs exhibit procoagulant and adhesion properties, and LOX-1 plays a key role in platelet–endothelium interaction. LOX-1 recognizes and binds activated platelets, which promote the release of endothelin-1 (ET-1), a potent vasoconstrictor agent with atherogenic properties [222]. When activated platelets bind to endothelial LOX-1, they induce ROS production and a consequent reduction of NO bioavailability [127]. Finally, the cross-linking of activated platelets by LOX-1 may stabilize thrombi and promote the thrombotic process [223].

## 18. Soluble LOX-1

**Figure 2:**
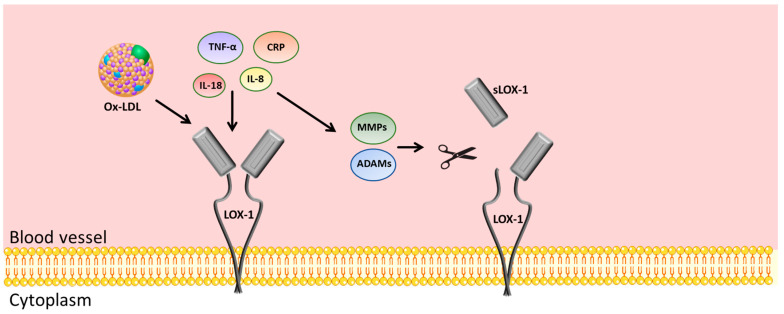
Generation of the soluble form of LOX-1 (sLOX-1) from the LOX-1 in the cell membrane. Various stimuli, including ox-LDLs, C-reactive protein (CRP), tumor necrosis factor (TNF)α, and interleukins such as IL-8 and IL-18 by the activation of matrix-degrading enzymes ADAMs and metalloproteinases (MMPs), induce the shedding of LOX-1 bound to the cellular membrane. sLOX-1 form is released into the bloodstream.

In recent years, LOX-1 has emerged as a promising novel biomarker in cardiovascular disease. Several studies have shown that circulating levels of soluble LOX-1 (sLOX-1) can be used as a diagnostic biomarker for an early diagnosis [224,225]. sLOX-1 is generated by ectodomain shedding and is released into the bloodstream, a process triggered by several stimuli, including ox-LDLs, C-reactive protein, tumor necrosis factor (TNF)α, and ILs such as IL-8 and IL-18, and is mediated by the activation of matrix-degrading enzymes ADAMs and metalloproteinases, as shown in Figure 2 [91]. The extent of the cleavage of the extracellular domain of LOX-1 reflects the endothelial dysfunction and oxidative stress status, both conditions associated with the onset and progression of atherosclerosis [226], as well as the destabilization of the atherosclerotic plaque in advanced atherosclerosis [227]. Studies have shown that sLOX-1 levels are increased in patients with acute coronary syndrome (ACS) [228,229]. The onset of ACS presumably depends on the perturbation of the balance between plaque instability and healing [230]. The activation of LOX-1 in this scenario contributes to the development of ACS. To determine the role of sLOX-1 and its implications in atherosclerosis development, Kraler et al. have demonstrated a positive correlation between sLOX-1 levels and plaque progression and/or fatal events in patients with ACS [229]. Specifically, they measured the levels of sLOX-1 in ACS patients compared with patients affected by chronic coronary syndrome and healthy subjects as controls. The results show that ACS patients presented elevated levels of circulating sLOX-1 in comparison with the other two groups of subjects [median 35.08 (15.75–73.44) vs. 2.00 (2.00–13.01) and 2.00 (2.00–6.93) pg/mL in ACS patients vs. chronic coronary syndrome and healthy subjects, respectively].

Comparing the levels of sLOX-1 with other specific biomarkers, such as troponin T (TnT) and heart-type fatty acid binding protein (H-FABP), which are high in ACS patients, has shown that sLOX-1 has higher sensitivity and specificity than both TnT and H-FABP [227]. Additionally, sLOX-1 has an advantage over other soluble biomarkers in that its peak is detectable at the early stage of ACS when TnT levels are not significantly elevated yet [227]. sLOX-1 levels may help predict prognosis after ACS as well. It has been observed that ACS patients who underwent percutaneous coronary intervention (PCI) and subsequently developed ACS recurrence and/or death had significantly higher levels of sLOX-1 than ACS patients who did not experience such events during the follow-up period [229]. Although PCI is a common approach for the revascularization of coronary arteries in ACS, this procedure can be associated with periprocedural complications such as PCI-related periprocedural myocardial infarction (RPMI). Studies have shown that high levels of sLOX-1 in the bloodstream can increase the risk of RPMI in patients undergoing PCI. This suggests that measuring sLOX-1 levels before the procedure can help identify patients at risk [91].

Additionally, it has been shown that sLOX-1 levels are critically associated with metabolic disorders like obesity, type 2 diabetes mellitus, and hypertension [231]. Patients with metabolic syndrome and coronary artery disease have been found to have higher levels of sLOX-1 compared to those without artery disease [232]. The upregulation of LOX-1 in metabolic diseases can contribute to the onset of cardiovascular complications [233,234]. Overall, sLOX-1 may serve as a potent early biomarker for predicting future cardiovascular events [91,228,235,236,237].

## 19. Clinical Use of LOX-1 Antibody

According to previous in vitro and in vivo studies, blocking the LOX-1 receptor could be an interesting mechanism to reduce inflammation and lipid-related risks in patients with cardiovascular disease [238]. Currently, human clinical studies on this topic are still in the developmental phase, with only one (NCT03654313) being in a more advanced stage. In this first-in-human, placebo-controlled study, 88 patients with type 2 diabetes were randomized to receive single ascending doses (10, 30, 90, 250, or 500 mg) or multiple ascending doses (90, 150, or 250 mg once monthly for 3 months) of MEDI6570, compared to placebo [239].

MEDI6570, a high-affinity human immunoglobulin G1 antibody targeting LOX-1, is designed to inhibit the binding of various lipid and inflammatory ligands to the LOX-1 receptor. The study aimed to evaluate the safety, efficacy, tolerability, immunogenicity, and pharmacokinetics of MEDI6570. The results have shown that MEDI6570 was well-tolerated, with no serious adverse events reported. The antibody also demonstrated high selectivity and low immunogenicity, and a reduction of the free sLOX-1 levels in a dose-dependent manner. Further, the pharmacokinetic analysis showed that the antibody effectively engaged with both soluble and membrane-bound LOX-1 targets, particularly at doses of 250 mg or higher. After administering three monthly doses of the antibody, the study found some regression of noncalcified plaque volume and a reduction of total plaque volume. However, the changes were not statistically significant. In conclusion, the study supports a monthly dosing administration of MEDI6570. Further research involving patients with coronary heart disease is necessary to confirm whether the antibody can reduce atherosclerosis and inflammation and eventually lead to long-term survival benefits [239].

## 20. Conclusions

**Figure 3:**
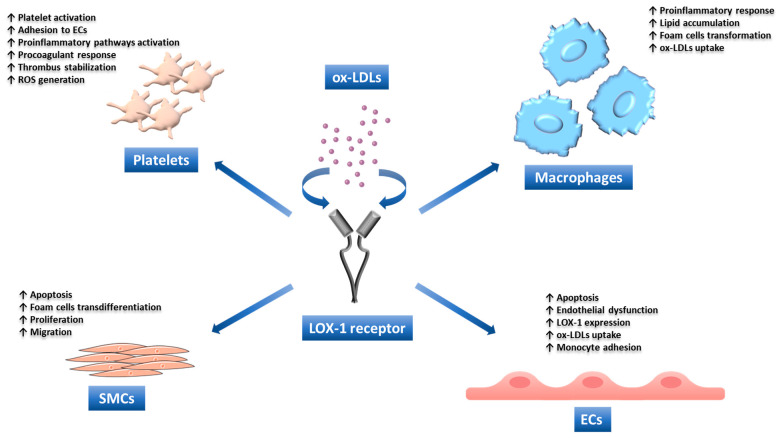
Schematic representation of the proatherogenic effects induced by the activation of LOX-1. Ox-LDL-mediated activation of the LOX-1 receptor induces endothelial dysfunction, promotes the recruitment of circulating monocytes, and activates SMCs and macrophage transformation in foam cells. The activation of platelets determines the onset of prothrombotic pathways, activating the procoagulant response and promoting thrombus stabilization. Comprehensively, these effects lead to the progression of atherosclerosis and promote the destabilization of the atherosclerotic plaque.

In the intricate environment of atherosclerotic plaque, the generation of ROS plays a crucial role in both the initiation and progression of atherosclerosis. ROS can oxidize LDLs and increase the expression of LOX-1, the receptor primarily involved in the binding and uptake of ox-LDL. The binding of ox-LDL to LOX-1 induces ROS generation, creating a vicious circle that amplifies the atherosclerotic process. While many studies have demonstrated the role of ROSs and ox-LDLs in atherosclerosis over time, the critical role of LOX-1 has only recently emerged. LOX-1 activation leads to several molecular pathways responsible for the atherosclerotic process, resulting in changes in the function of various cell types. This can cause endothelial dysfunction, muscle-cell proliferation, apoptosis, monocyte recruitment, and foam-cell formation (Figure 3). High levels of sLOX-1 have been found in various pathological conditions associated with cardiovascular diseases, suggesting that sLOX-1 may serve as a potential biomarker for cardiovascular disease [133,197,240,241,242]. From a therapeutic perspective, blocking LOX-1 with natural or synthetic compounds may represent an attractive approach to counteracting the atherosclerotic process and its associated conditions.

## Figures and Tables

**Table 1:** LOX-1 inhibitors and their role in cardiovascular diseases.

Compound	Nature of Compound	Role in Cardiovascular Diseases	Reference
Curcumin	Natural polyphenol	Ameliorates dyslipidemia Increases NO levels Improves endothelial function Reduces PPAR-γ and CD36 expression in macrophages Increases cholesterol efflux through ABCA1 Blocks superoxide production Reduces LOX-1 expression	Singh, L. et al. [149] Panahi, Y. et al. [150] DiSilvestro, R.A. et al. [151] Oliver, J.M. et al. [152] Ramaswami, G. et al. [153] Lee, H.S. et al. [154]
Tanshinone II-A	Natural diterpenoid naphthoquinone	Inhibits LOX-1 expression in murine macrophages and atherosclerotic lesions	Xu, S. et al. [155]
Resveratrol	Natural compound	Lowers blood pressure Improves endothelial function Increases NO levels Inhibits platelet aggregation Reduces the synthesis of inflammatory prostaglandins Suppresses LOX-1 expression	Iqbal, I. et al. [157] Dyck, G.J.B. et al. [158] Guo, R. et al. [159]
Bergamot-derived polyphenolic fraction	Natural polyphenol	Decreases LOX-1 expression in polymorphonuclear leukocytes of hyperlipidemic patients In combination with rosuvastatin enhances its antioxidant activity, normalizes the serum lipemic profile of hyperlipidemic patients, and increases the expression and phosphorylation of protein kinase B Vasoprotective and anti-proliferative effects Reduces vascular damage	Gliozzi, M. et al. [160]
Ginkgolide B	Natural compound	Protects from endothelial dysfunction Inhibits LOX-1 expression Reduces lipid and cholesterol accumulation	Feng, Z. et al. [161]
BI-0115	Synthetic small molecule	Inhibits ox-LDL internalization Reduces edema formation and infarcted area in ischemic stroke model Increases barrier integrity of brain microvascular endothelial cells exposed to ischemia–reperfusion	Schnapp, G. et al. [162] Arkelius, K. et al. [163]
Lovastatin	Statin	Reduces cholesterol levels in plasma membrane Reduces ox-LDL uptake mediated by LOX-1 Prevents ox-LDL-induced apoptotic phenotype	Matarazzo, S. et al. [164]
Losartan	AT1 receptor blocker	Reduces LOX-1 upregulation and ox-LDL internalization	Morawietz, H. et al. [167] Li, D.Y. et al. [168]
Quinapril	ACE inhibitor	Decreases the levels of LOX-1, p22phox, p47phox and gp91phox in the left ventricle of Dahl rats	Kobayashi, N. et al. [169]
Anti-LOX-1 monoclonal antibodies	Monoclonal antibody	Reduces the formation of ROS induced by ox-LDL Increases the production of intracellular NO in HUVEC Prevention of LOX-1 upregulation mediated by ischemia/reperfusion injury in Sprague–Dawley rat model Reduces MMP-1 and adhesion molecule expression Reduces leukocyte recruitment and infarct size in ischemia–reperfusion model Reduced lipid deposits in mesenteric arteries of stroke-prone hypertensive rats Prevents coronary artery endothelial dysfunction, reduces superoxide production, increases eNOS expression, and restores arteriolar dilatation in ApoE^−/−^ mice	Li, D. et al. [170] Nakano, A. et al. [171] Xu, X. et al. [172]
